# Implementing Best Practice Guidelines under the Best Practice Spotlight Organisation: Facilitators and Barriers for Nurses: A Delphi Study

**DOI:** 10.3390/nursrep14030185

**Published:** 2024-09-20

**Authors:** Noel Rivas-González, Belén Martín-Gil, Mercedes Fernández-Castro

**Affiliations:** 1 Continuing Education Department, Valladolid University Clinical Hospital, 47011 Valladolid, Spain; nrivas@saludcastillayleon.es; 2 Department of Nursing Care Information Systems, Valladolid University Clinical Hospital, 47011 Valladolid, Spain; bmartingi@saludcastillayleon.es; 3 Research Support Unit, Valladolid University Clinical Hospital, 47011 Valladolid, Spain

**Keywords:** evidence-based practice, competence, barrier, facilitator, implementation, nursing

## Abstract

The implementation and sustainability of evidence-based practice (EBP) is a complex process. Best practice guidelines (BPGs) can facilitate the translation of knowledge from theory into practice, but they require working groups to adapt recommendations and encourage adherence to them. **The aim** of this study was to identify facilitators and barriers of BPGs in the Registered Nurses’ Association of Ontario^®^, within the framework of Best Practice Spotlight Organisations^®^ (BPSOs^®^), in a hospital setting. **Methods:** This study was conducted using the modified Delphi method (two rounds) with a cohort of BPSO^®^ Best Practice Champions. The facilitators and barriers included in the first round were identified through a bibliographic search. The degree of concordance was determined in the second round using the highest mean resulting values. **Results:** A total of 44 nurses responded, with a mean age of 42.57 ± 8.563. The facilitators included the following: work environment; working in a hospital that encourages research; and dedicating time to EBP (*p* < 0.001). The barriers included the following: excessive workload; professional mobility (*p* < 0.001); and lack of time (*p* = 0.002). **Implications for practice**: As such, it is necessary to approach human resource management by implementing new policies that guarantee systemic sustainability. The BPSO^®^ programme is an excellent framework for promoting the translation of theoretical knowledge into practice and effecting institutional change.

## 1. Introduction

Work environments that are poorly equipped to promote the proper development of nursing care may have an impact on patient health outcomes [1,2].

Evidence-based practice (EBP), the result of translating theoretical knowledge into clinical practice, can aid nursing staff in achieving healthcare excellence. It facilitates the decision-making process, if and when nurses are properly informed of and involved in this process [3].

The implementation and sustainability of EBP in healthcare settings is a complex process that requires a solid foundation. The international implementation programme for best practice guidelines (BPGs), the Best Practice Spotlight Organisation^®^ (BPSO^®^) network, seeks to transform the field of nursing through knowledge, improving quality of care by implementing BPGs developed by the Registered Nurses’ Association of Ontario^®^ (RNAO^®^) as per the Knowledge to Action method, with an international data system for evaluating results. This methodology comprises six fundamental phases: (1) problem identification; (2) adaptation to the local context; (3) evaluation of facilitators and barriers; (4) adaptation and implementation of interventions; (5) monitoring and evaluation of outcomes; and (6) sustainability. It is consistently applied to the implementation of each guideline. The BPSO programme originated in Ontario, Canada, in 1999, with the aim of enhancing nurses’ competencies and translating evidence into clinical practice, thereby achieving results at institutional, patient, and healthcare system levels by influencing health policies. The demand has been such that, to date, 42 clinical guidelines and 12 guidelines for healthy work environments have been developed, which are being implemented in over 1000 healthcare and academic institutions internationally [4]. These institutions are selected through a competitive process: centres submit their proposals to implement and evaluate RNAO^®^ BPGs over a three-year period, during which time they are considered to be candidates. Subsequently, they continue the implementation and evaluation process, formalising new agreements as designated BPSOs^®^, with sustainability and participation in the programme being reassessed every two years. In Spain, this process is led by the Healthcare and Health Services Research Unit at the Carlos III Institute of Health under the Spanish Ministry of Science, Innovation, and Universities, and in collaboration with the CECBE (the Spanish Centre for Evidence-Based Healthcare), which signed an agreement with RNAO in 2010. The programme is led by nurses, establishing an institutional implementation committee and a network of professionals who receive systematic training to carry out their task as champions to drive the programme forward within their chosen field of implementation. These champion nurses are the cornerstone of both the rolling-out and sustainability of the programme within their institutions under this framework [4,5,6,7].

Several studies have identified and described elements that help facilitate the implementation of EBP in general, as well as barriers that hinder this process. Facilitators include managerial support, academic training, structured EBP training, organisational EBP culture, and the existence of a critical mass of EBP mentors [8,9,10]. The most frequently mentioned barriers include lack of time, rejection of statistics, preference for intuitive and experience-based methods, insufficient resources, lack of solid leadership, and the lack of knowledge [8,10,11,12,13].

Regarding the BPSO^®^ framework, this field has not been thoroughly researched. The authors of a study conducted in Australia described a solid implementation and programme execution plan, as well as support from management, as facilitators. The barriers they found included the following: professional motivation, difficulty finding cover on days staff wished to attend a training course, and the lack of institutional commitment to see through the programme [14].

The evaluation of the implementation of the BPSO^®^ programme in Spain was positive regarding nurse perceptions of work environments, available resources, and the possibility for professional development [15].

The implementation of BPGs in a hospital setting entails the involvement of active working groups that will tailor recommendations to their specific setting, and, most importantly, act as “guardians” to ensure adherence to the plan, recording of activity, and the training of newly hired nursing staff. Between 2019 and 2024, there have been several working groups, all strategically composed to meet the implementation needs of each of the BPGs. At the time of writing, June 2024, the hospital is in the sustainability phase of three of the BPGs, and the implementation phase of a fourth, boasting 50 active champions spread across the various hospital departments. This study aims to identify the facilitators and barriers identified by this cohort of 50 best practice champion nurses, all actively involved in the implementation of the BPGs of the RNAO^®^, within the framework of the BPSO^®^ strategy, in a hospital setting.

## 2. Materials and Methods

### 2.1. Study Design, Setting, and Sampling

To reach consensus among the study population, the modified Delphi method was proposed (two rounds of questions). The study group consisted of 50 nurses who had attended the eight-hour “Best Practice Champions Workshop” and who, at the time of the study, were actively involved in working groups to implement and/or upkeep BPG recommendations by creating materials and protocols, assessing indicators or hosting information sessions in their individual departments. The research team spearheaded the implementation of the BPSO programme at our institution, delivering training to the champions and overseeing the entire process. Close collaboration with the 50 professionals was a priority, focusing on understanding their perspectives on the barriers and facilitators in implementing best practices within the BPSO framework. These insights will guide the development of strategies to further streamline the process. The study population was interacted with during working group meetings.

This study was conducted in Valladolid (Spain) at a tertiary care hospital in the Castile and León Health System with 777 beds and a staff of 918 nurses.

### 2.2. Instruments

To identify facilitators and barriers, an integrative review was conducted to provide a comprehensive overview of the subject. A bibliographic search was carried out in the PubMed and Cochrane Library databases using the following MeSH terms: Evidence-based practice; Competence; Barrier; Facilitator; Implementation; Healthcare; and Nurses. Publications had to have been published in the last five years in either English or Spanish, and had to include an analysis of barriers and facilitators encountered by nurses when implementing EBP. Studies conducted in academic settings or with nursing students as the study population were excluded. All types of articles were considered, with the final search carried out on 30 November 2023. After the systematic search, a screening of the retrieved records was performed by reviewing titles and abstracts, selecting those that met the inclusion criteria. Data extraction tables were then created, including information on the title, author, year, study type, and key findings. A total of 23 studies met the inclusion criteria, from which 35 facilitators and 33 barriers were identified. These findings informed the first round of questions of the Delphi method. The research team designed 35 questions, one for each facilitator identified, and other 33 questions related to the identified barriers. A pilot test was conducted with a convenience sample of 10 nurses to assess question clarity, with no changes suggested to the items.

Nurses were required to rate how much they agreed with each item on a scale of 1 to 5, with 1 being “Strongly disagree”, 2 being “Disagree”, 3 being “Neither agree nor disagree”, 4 being “Agree”, and 5 being “Strongly agree”. When moving onto the second round of the Delphi technique, the questionnaire was redesigned, ordering items based on the mean scores that were obtained in the first round, from highest to lowest. This time, the questionnaire asked about agreement with the obtained score, from 1 to 5, with 1 being “Strongly disagree” and 5 being “Strongly agree”. Each round was conducted with an interval of one month between them. The rounds were conducted in March and April 2024 and questionnaires were sent to participants through mailing lists of work email addresses, having previously contacted the study population to explain the aim of the study and request their anonymous and voluntary participation.

Sociodemographic and work-based variables were also included.

Consensus on facilitators and barriers was reached using the highest mean values obtained in the first round that also had the highest degree of concordance in the second round. The consensus threshold was set at a mean value of ≥3.50 for both barriers and facilitators.

### 2.3. Ethical Considerations

The anonymity and confidentiality of the surveyed participants was maintained at all times, as per Regulation (EU) 2016/679 of the European Parliament and of the Council of 27 April 2016 (GDPR), enshrined in Spanish law under the Spanish Organic Law 3/2018 of 5 December, on Personal Data Protection and Guarantee of Digital Rights. This study was approved by the Valladolid Ethics Committee for Research with medicinal products (ECRmp) involving humans under the reference code PI-24-257-C.

### 2.4. Statistical Analysis

The statistical analysis was conducted using the IBM SPSS Statistics software (v. 24; IBM, Armonk, NY, USA). Quantitative variables were expressed as means and standard deviations, and qualitative variables as frequency distributions. Pearson’s chi-squared test was used to analyse associations between qualitative variables, while Student’s *t*-test was used to compare quantitative variables. Pearson’s chi-squared test was also used to identify the degree of concordance in the second round of the Delphi method. Values of *p* < 0.05 were considered as statistically significant.

## 3. Results

In the first round, 44 nurses responded (88% of the study population), while 39 responded in the second round (78%). The mean respondent age was 42.57 (SD = 8.563) with an average of 18.23 (SD = 8.118) years of professional experience. Respondents reported having read an average of four scientific articles in the last month (SD = 6.324). The majority were women (93.2%) holding a permanent position (61.4%) in the field of healthcare (88.6%). Only 6% reported having had more than 50 h of EBP training, despite 63.7% holding a postgraduate degree and 56% having been best practice champions for more than one year (Table 1).

Table 2 and Table 3 show the list of facilitators and barriers perceived by nurses that were obtained in the first round, sorted by the highest mean value (above 3.5). The degree of concordance from the second round is also shown comparatively in two columns: one column lists responses of 4 “Agree” or 5 “Strongly agree”, while the other lists responses of 1 “Strongly disagree”, 2 “Disagree”, or 3 “Neither agree nor disagree”.

“Work environment” (mean = 4.55; SD = 0.82), “Working in a hospital that promotes research” (mean = 4.36; SD = 0.83), and “Free time during the workday to dedicate to implementation-related tasks (protocols, materials, etc.)” (mean = 4.34; SD = 1.12) were the highest scoring facilitators on average and had a significantly higher degree of concordance in the agree vs. disagree percentages at *p* < 0.001. There was no consensus on the order of importance of some items, such as the existence of “Forums to discuss cases, critical reading or research projects” (mean = 3.82; SD = 0.922) and having completed “Postgraduate studies” (mean = 3.50; SD = 0.849), each of which with an insignificant degree of concordance at *p* = 0.150 and *p* = 0.262, respectively (Table 2).

In terms of barriers, “Excessive workload” (mean = 4.43; SD = 0.728), “Rotations and staff transfers” (mean = 4.34; SD = 0.680), and “Lack of time” (mean = 4.32; SD = 0.708) were the items with the highest mean scores and highest degree of concordance between group members, with *p* < 0.001 in the first two and *p* = 0.002 in the third. “Lack of authority to change practice” (mean = 3.73; SD = 0.973), “Aversion to statistics” (mean = 3.59; SD = 1.226), “Lack of forums/areas to discuss evidence with colleagues” (mean = 3.55; SD = 0.848), and “Nursing culture governed by hierarchical rules” (mean = 3.55; SD = 1.130) were all items with a low degree of consensus on their position (*p* > 0.05) (Table 3).

## 4. Discussion

Identifying barriers and facilitators during the implementation process of the recommendations detailed in BPGs, as per the BPSO© programme, is necessary for both this programme’s longevity over time as well as its solidification as a cultural institution. This process is considered a key step in defining support strategies that promote change in clinical practice [6,14].

During the evaluation process, it was found that the facilitators agreed on by best practice champion nurses were primarily related to the institutional framework. Nurses felt that management ought to promote a work environment that encourages implementation and allows for time during the workday to dedicate to implementation-related tasks; support from superiors; involvement of other professional categories; better recognition of research activities; and alignment of EBPs with strategic and institutional goals. This should be a priority for management, given that adopting EBPs improves not only quality of care and healthcare outcomes for patients, but also professional competence in reflective practice, thereby aiding in their professional development—a conclusion echoed by other studies undertaken on the same topic [16,17,18]. Furthermore, management should consider factors implicit to effecting change that are related to organisational aspects, leadership, groups of interest, structures, internal communications, and available resources. Consequently, the implementation of best practice guidelines requires thorough planning of comprehensive strategies to effect progressive cultural change on a professional and institutional level [19].

In this regard, leadership is a key facilitator of the programme, as several previous works have already noted and whose results coincide with those of this study. Autonomy in the decision-making process and the security offered by the best available evidence transform nurses into exemplary leaders of clinical care, which directly translates into professional empowerment and development [20,21]. As a result, this contributes to the creation of better-suited working conditions, leading to better safety results for both nurses and patients alike, which, in turn, increases workplace satisfaction, as identified by other authors [10,22]. As their field of clinical practice improves, nurses realise that their work is being recognised, leading to a better perception of their working environment compared to nurses who are not best practice champions. The proactive and participative approach of the programme improves interdepartmental relationships. Furthermore, involvement in organisational decision-making and hospital policymaking have been shown to be of significant importance, generating a feeling of belonging in the institution. This sentiment has previously been studied by the authors, in which the positive repercussions that the implementation of BPG recommendations has on clinical practice were discussed [23]. These circumstances may grant champion nurses the role of agents of change in daily practice, in contrast to other studies wherein the lack of authority to make changes has been described as a barrier [10].

It must be noted that there were some discrepancies, specifically that no degree of concordance was reached on whether “Postgraduate studies” was a facilitator or not. This may be due to the fact that a large proportion of nurses in our sample already had a postgraduate qualification and therefore prioritised extrinsic or institutional factors over intrinsic or personal factors. While it is true that the academic background of nursing may not be comparable one-to-one with other sciences, the efforts of today’s professionals have given rise to both professional and academic recognition of the profession, empowering the nursing discipline to implement and follow EBPs with increasing ease, and also tackle the necessary challenges to complete this implementation—though this does not mean they are exempt from future challenges and hurdles [16].

Regarding the barriers perceived by our study population, of particular note were the challenges related to the management of human resources in the Spanish healthcare system and the lack of nurses. Excessive workload, rotations and staff transfers, lack of time, or burnout and work dissatisfaction are, without a doubt, significant areas for improvement, alongside unstable work contracts and an ageing population with increased care needs—all of which require new policies that will guarantee the sustainability of the healthcare system [24,25].

Despite clearly being considered significant barriers, as with other studies on the topic, in this study, there was no agreement reached regarding the order of “Lack of authority to change practice” and “Nursing culture governed by hierarchical rules”. However, these barriers are considered a recurring theme in nursing practice, requiring close, interprofessional collaboration to develop strategies to combat the situation [26].

The BPSO programme was selected by the WHO European Region in its compendium report Lessons from transforming health services delivery: compendium of initiatives in the WHO European Region for its impact as a best practice [27]. This is particularly relevant for identifying the barriers and facilitators reported by the nurses involved in its implementation and has significant implications for practice, encouraging the design of effective strategies for care management and setting the stage for future research on the effectiveness of these strategies. The Valladolid University Clinical Hospital became the first tertiary healthcare facility in the Castile and León region to be designated a BPSO in 2022, making the identification of barriers and facilitators encountered by their champions an example for other institutions in the region to consider during their candidacy period.

All of the aforementioned aspects invite active input from management as a key part of designing institutional policies to promote the main facilitators and minimise the barriers perceived by nurses when implementing evidence-based practice recommendations.

The main limitation is related to the Delphi method itself, particularly the tendency of some participants, after the first round, to be tempted to adjust their responses to match the group’s overall scoring, rather than maintaining their own independent judgement. Moreover, this study was conducted in a very specific setting and in a single hospital, which may make it difficult to generalise the results.

## 5. Conclusions

The barriers and facilitators perceived by best practice champion nurses mainly lean towards extrinsic or institutional factors over intrinsic or personal factors. Barriers such as excessive workload, rotations and staff transfers, lack of time, lack of incentives/remuneration, and staff shortages, as well as facilitators such as a supportive work environment, working in a hospital that promotes research, or having free time during the workday to dedicate to implementation tasks (e.g., protocols, materials), all fall under the responsibility of management. As such, management should plan comprehensive strategies that effect gradual change in the culture of the organisation. The BPSO^®^ programme is the best-suited framework to translate theoretical knowledge into clinical practice and bring about the required institutional change.

## Figures and Tables

**Table 1:** Description of the study sample.

Variable		*n*	%
Sex	Male	3	6.8%
	Female	41	93.2%
Employment status	Locum	17	38.6%
	Permanent	27	61.4%
The field in which they carry out their role is	Healthcare	39	88.6%
	Teaching	2	4.5%
	Management	6	13.6%
Postgraduate level	Specialisation	12	27.3%
	Master’s	16	36.4%
	None	16	36.4%
Specific Training in EBP and/or Research Methodology in recent years	From 8 to 50 h	14	31.8%
	More than 50 h	6	13.6%
When did they join the Best Practice Champions network	In the last year	18	20.5%
	In the last one to three years	14	31.8%
	More than three years ago	12	25.0%

**Table 2:** Mean scores of facilitators obtained in the first round and a comparison of agreement vs. disagreement with the scores obtained in the second round.

Facilitators	First Round (n = 44)		Second Round (n = 39)				
			Disagree (1,2,3)		Agree (4,5)		
	Mean	Standard Deviation	Disagree N	%	Agree N (%)	%	*p*-Value
1-Work environment	4.55	0.820	7	17.9%	32	82.1%	<0.001
2-Working in a hospital that promotes research	4.36	0.838	5	12.8%	34	87.2%	<0.001
3-Free time during the workday to dedicate to implementation-related tasks (protocols, materials, etc.)	4.34	1.119	8	20.5%	31	79.5%	<0.001
4-Support from superiors	4.30	0.954	8	20.5%	31	79.5%	<0.001
5-Better recognition of research activity	4.27	0.949	8	20.5%	31	79.5%	<0.001
6-Support from site management	4.25	0.918	6	15.4%	33	84.6%	<0.001
7-Guidance when developing research projects	4.23	1.008	6	15.4%	33	84.6%	<0.001
8-Evidence-based practice aligns with site objectives	4.20	0.795	7	17.9%	32	82.1%	<0.001
9-Involvement of all professional categories	4.20	0.851	7	17.9%	32	82.1%	<0.001
10-Suitable environment	4.20	0.878	9	23.1%	30	76.9%	0.001
11-Having a colleague who is an evidence-based practice champion	4.20	0.878	6	15.4%	33	84.6%	<0.001
12-Nurse leadership	4.16	0.939	10	25.6%	29	74.4%	0.002
13-A rewards system for motivated nurses	4.16	1.119	16	41.0%	23	59.0%	0.262
14-Institutional support	4.14	0.852	9	23.1%	30	76.9%	0.001
15-Holding sufficient authority to implement BPG recommendations	4.11	0.868	10	25.6%	29	74.4%	0.002
16-Dissemination of works resulting from implementation activities, conferences, or publications	4.07	0.900	11	28.2%	28	71.8%	0.006
17-Flexibility to implement practice	4.05	0.861	11	28.2%	28	71.8%	0.006
18-Communication and training within the organisation based on the results of research	4.05	0.987	12	30.8%	27	69.2%	0.016
19-Retention of talent by the organisation	4.05	1.099	15	38.5%	24	61.5%	0.150
20-Evidence-based organisational policies and protocols	4.02	0.902	15	38.5%	24	61.5%	0.150
21-Access to scientific resources at work	4.02	0.927	13	33.3%	26	66.7%	0.037
22-Access to computers and the Internet at work	4.02	1.171	9	23.1%	30	76.9%	0.001
23-Sufficient resources	4.00	0.988	7	17.9%	32	82.1%	<0.001
24-Ongoing education in evidence-based practice	3.95	1.056	12	30.8%	27	69.2%	0.016
25-Funding	3.95	1.099	14	35.9%	25	64.1%	0.078
26-Having mentors	3.93	0.900	13	33.3%	26	66.7%	0.037
27-Collaborative relationships of mutual support with other professionals	3.91	1.053	8	20.5%	31	79.5%	<0.001
28-Research training	3.91	1.053	6	15.4%	33	84.6%	<0.001
29-Collaboration between the hospital and the university	3.86	0.878	8	20.5%	31	79.5%	<0.001
30-A trusting professional relationship	3.84	0.987	8	20.5%	31	79.5%	<0.001
31-Forums to discuss cases, critical reading, or research projects	3.82	0.922	15	38.5%	24	61.5%	0.150
32-Workplace culture	3.75	1.014	11	28.2%	28	71.8%	0.006
33-Better administrative support	3.73	1.169	12	30.8%	27	69.2%	0.016
34-Postgraduate studies	3.50	0.849	16	41.0%	23	59.0%	0.262

**Table 3:** Mean scores of barriers obtained in the first round and a comparison of agreement vs. disagreement with the scores obtained in the second round.

Barriers	First Round (n = 44)		Second Round (n = 39)				
			Strongly Disagree; Disagree; Neither Agree nor Disagree		Agree or Strongly Agree		
	Mean	Standard Deviation	N	%	N	%	*p*-Value
1-Excessive workload	4.43	0.728	8	20.5%	31	79.5%	<0.001
2-Rotations and staff transfers	4.34	0.680	6	15.4%	33	84.6%	<0.001
3-Lack of time	4.32	0.708	10	25.6%	29	74.4%	0.002
4-Lack of incentive/remuneration	4.30	0.823	12	30.8%	27	69.2%	0.016
5-Lack of staff	4.23	1.008	8	20.5%	31	79.5%	<0.001
6-Burnout and work dissatisfaction	4.20	0.668	13	33.3%	26	66.7%	0.037
7-Insufficient funding for healthcare research projects	4.07	0.789	14	35.9%	25	64.1%	0.078
8-Resistence to change	3.93	1.108	11	28.2%	28	71.8%	0.006
9-Culture of “we’ve always done it this way”	3.93	1.129	9	23.1%	30	76.9%	0.001
10-Insufficient time to find and read research projects	3.91	1.096	13	33.3%	26	66.7%	0.037
11-Lack of authority to change practice	3.73	0.973	17	43.6%	22	56.4%	0.423
12-Organisational factors	3.64	0.750	10	25.6%	29	74.4%	0.002
13-Lack of knowledge to interpret scientific evidence	3.61	1.039	13	33.3%	26	66.7%	0.037
14-Aversion to statistics	3.59	1.226	16	41.0%	23	59.0%	0.262
15-Poorly defined priorities	3.55	0.875	14	35.9%	25	64.1%	0.078
16-Lack of forums/areas to discuss evidence with colleagues	3.55	0.848	16	41.0%	23	59.0%	0.262
17-Nursing culture governed by hierarchical rules	3.55	1.130	15	38.5%	24	61.5%	0.150

## Data Availability

The data presented in this study are available upon request from the corresponding author.

## Public Involvement Statement

There was no public involvement in any aspect of this research.

## Guidelines and Standards Statement

This manuscript was drafted against the STROBE (The Strengthening the Reporting of Observational Studies in Epidemiology) for observational research.
